# Effect of Tadalafil on Seizure Threshold and Activity of Antiepileptic Drugs in Three Acute Seizure Tests in Mice

**DOI:** 10.1007/s12640-018-9876-4

**Published:** 2018-02-09

**Authors:** Katarzyna Socała, Dorota Nieoczym, Mateusz Pieróg, Elżbieta Wyska, Małgorzata Szafarz, Urszula Doboszewska, Piotr Wlaź

**Affiliations:** 1 Department of Animal Physiology, Institute of Biology and Biochemistry, Maria Curie-Skłodowska University, Akademicka 19, 20-033 Lublin, Poland; 2 Department of Pharmacokinetics and Physical Pharmacy, Jagiellonian University Medical College, Kraków, Poland

**Keywords:** Tadalafil, PDE5 inhibitors, Seizure threshold, Antiepileptic drugs, Mice

## Abstract

Tadalafil, a selective phosphodiesterase type 5 inhibitor, is a long-acting oral agent for the treatment of erectile dysfunction of multiple etiologies. Although generalized tonic-clonic seizures were reported in a healthy man after taking tadalafil, the influence of tadalafil on seizure susceptibility has not been studied so far. Therefore, the aim of the present study was to investigate the effect of tadalafil on seizure threshold as well as on the activity of some first- and second-generation antiepileptic drugs in three acute seizure tests in mice. The obtained results showed that tadalafil, at the highest dose tested (20 mg/kg), significantly decreased the threshold for the first myoclonic twitch in the intravenous pentylenetetrazole (i.v. PTZ) seizure test. It did not affect the threshold for generalized clonic seizure and forelimb tonus in the i.v. PTZ, for tonic hindlimb extension in the maximal electroshock seizure threshold test, and for psychomotor seizure in the 6-Hz-induced seizure threshold test. Tadalafil did not alter the anticonvulsant activity of any of the studied antiepileptic drugs in electrically induced seizure tests. Interestingly, tadalafil potentiated the anticonvulsant activity of clonazepam and decreased the anticonvulsant activity of oxcarbazepine in the i.v. PTZ test. These interactions were pharmacodynamic in nature, as tadalafil did not alter clonazepam and oxcarbazepine concentrations both in serum and brain tissue. Furthermore, neither tadalafil alone nor its combinations with the studied antiepileptic drugs produced any significant impairment of motor coordination (assessed in the chimney test), muscular strength (investigated in the grip-strength test), and long-term memory (assessed in the passive avoidance task). In conclusion, tadalafil may increase the risk of myoclonic seizure and decrease the anticonvulsant efficacy of oxcarbazepine. Further studies are warranted to evaluate the safety of tadalafil usage in patients with epilepsy.

## Introduction

Erectile dysfunction, which is defined as a persistent or recurrent inability to obtain or maintain penile erection during sexual intercourse, is a highly prevalent disorder that is expected to reach 322 million cases by the year 2025 (McKinlay 2000). It is closely associated with aging, lifestyle, and comorbid diseases such as cardiovascular disease, hypertension, diabetes mellitus, depression, or neurological disorders including epilepsy (Wyllie 2005). Erectile dysfunction has been estimated to occur in up to 65% of men suffering from epilepsy and may be related to endocrine system abnormalities caused by epileptic discharges or antiepileptic drug treatment, psychiatric comorbidities, and psychosocial problems (Hamed et al. 2013; Vieira et al. 2015).

Oral drug treatment with phosphodiesterase type 5 (PDE5) inhibitors is currently first-line therapy for erectile dysfunction. The three widely available drugs from this class are sildenafil citrate (Viagra®, Pfizer, USA), vardenafil (Levitra®; Bayer AG, Germany), and tadalafil (Cialis®; Eli Lilly and Company, USA). They share a similar mode of action. Briefly, sexual arousal stimulates the nitric oxide (NO)/cyclic guanosine 3′,5′-monophosphate (cGMP) signaling pathway in the corpus cavernosum smooth muscle cells. PDE5 inhibitors increase the intracellular level of cGMP by inhibiting its breakdown, which improves smooth muscle relaxation and prolongs penile erection (Coward and Carson 2008; Huang and Lie 2013).

Although no clinical trials focusing on the effects of sildenafil, vardenafil, or tadalafil on seizure activity were performed, these medications are generally considered as safe and effective therapy in patients with epilepsy and co-existing erectile dysfunction (Harden 2002; Matos et al. 2012). Nevertheless, the safety of PDE5 inhibitors in epileptic patients is questionable (Matos et al. 2012). Generalized tonic-clonic seizures were first reported in two healthy men after taking sildenafil (Gilad et al. 2002). Then, epileptic seizures were observed in two men taking vardenafil (Koussa et al. 2006; Striano et al. 2006). Proconvulsant effect of sildenafil was also shown in the intravenous pentylenetetrazole (i.v. PTZ) and bicuculline seizure tests in mice (Montaser-Kouhsari et al. 2011; Nieoczym et al. 2010b; Riazi et al. 2006). Sildenafil produces, however, contradictory effects in animal models of seizure and epilepsy. Its anticonvulsant action was observed in the maximal electroshock seizure threshold (MEST) test (Nieoczym et al. 2010a), in the 6-Hz-induced seizure test in mice (Nieoczym et al. 2013), and in the amygdala kindling in rats (Nieoczym et al. 2010b). Moreover, several studies showed that sildenafil potentiated the activity of some antiepileptic drugs in animal models of seizures (Nieoczym et al. 2010a, 2012a, 2012b, 2013). However, pharmacokinetic interactions of sildenafil with carbamazepine and valproate raise concern about the safety of its usage in combination with these antiepileptic drugs (Nieoczym et al. 2010a). It seems that sildenafil may affect seizure susceptibility and the activity of antiepileptic drugs. Thus, caution should be taken when prescribing this PDE5 inhibitor in patients with epilepsy (Matos et al. 2012; Nieoczym et al. 2010a).

Tadalafil has a markedly different molecular structure than sildenafil and vardenafil, and these structural differences have implications for the selectivity and pharmacokinetics of the three PDE5 inhibitors. Sildenafil and vardenafil bind not only to PDE5, but they are also quite potent PDE6 inhibitors. In comparison, tadalafil is highly selective for PDE5, has low affinity for PDE6, and potently inhibits PDE11—an enzyme with an unclear physiological function (Coward and Carson 2008; Ferguson and Carson 2013). The most striking difference among the three PDE5 inhibitors is their duration of action. Specifically, in humans, the half-lives of sildenafil and vardenafil are at about 4 h, while tadalafil has much longer half-life of approximately 17.5 h (Huang and Lie 2013).

Tadalafil’s longer half-life may pose a risk of long-lasting side effects including increased seizure susceptibility (Matos et al. 2012). It was shown that two days after single administration, tadalafil produced detectable electroencephalographic changes in up to 34% of healthy men (Okuyucu et al. 2009). In animal studies, it accelerated seizure development in rats exposed to hyperbaric oxygen (Demchenko et al. 2009). Furthermore, tonic-clonic seizures in a healthy young man following tadalafil intake were reported (Calabro et al. 2013). Thus, it seems that tadalafil may exert proconvulsive effects. Considering the above data, we aimed to evaluate the acute effect of tadalafil on the seizure threshold in three seizure tests in mice, i.e., in the i.v. PTZ infusion test, the MEST test, and the 6-Hz-induced psychomotor seizure test. In addition, the longer duration of action of tadalafil may increase the risk for drug interactions. Since there is no data on the interactions between tadalafil and antiepileptic drugs, we also investigated the influence of tadalafil on the activity of several first- and second-generation antiepileptic drugs.

## Materials and Methods

### Animals

The experiments were performed on 1028 naïve male albino Swiss mice weighing 25–30 g. The animals were purchased from a licensed breeder (Laboratory Animals Breeding, Ilkowice, Poland) and housed in groups of 8 in Makrolon cages (37 cm × 21 cm × 14 cm) under strictly controlled laboratory conditions (temperature maintained at 21–24 °C, relative humidity at 45–65%) with an artificial 12/12 h light/dark regime (light on at 6:00 a.m.). A nutritionally balanced rodent chow diet (Murigran, Agropol S.J., Motycz, Poland) and tap water were continuously available. Animals were used in the study after at least one week of acclimatization. All experiments were performed between 8:00 a.m. and 3:00 p.m., after a minimum 30-min adaptation period to the conditions kept in the experimental room. The animals were randomly assigned to the experimental groups. Each animal was used only once.

The study was performed under experimental protocols approved by the Local Ethical Committee in Lublin. Housing and experimental procedures were conducted in accordance with the European Union Directive of 22 September 2010 (2010/63/EU) and Polish legislation acts concerning animal experimentation. All efforts were made to minimize animal suffering as well as the number of animals used in the study.

### Drugs

**Fig. 1:**
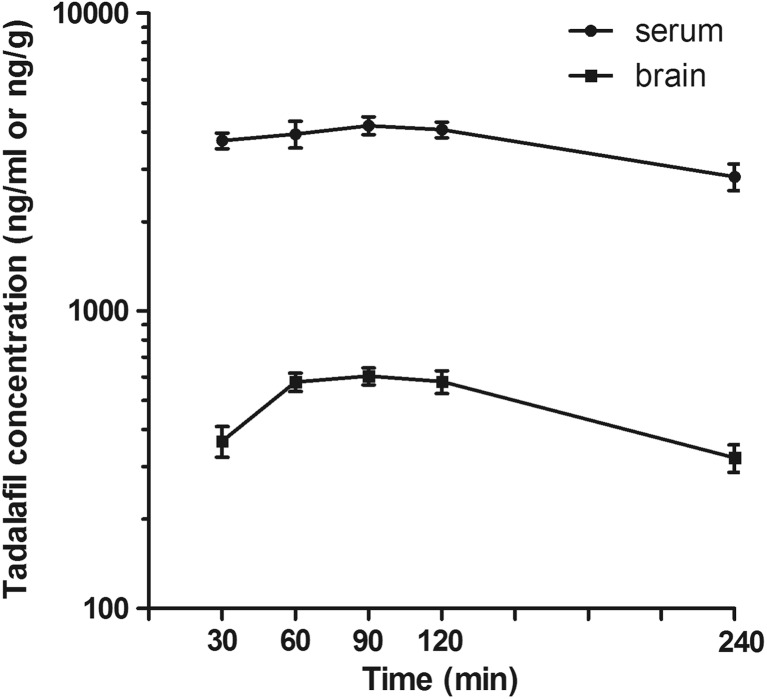
Serum and brain concentrations of tadalafil in mice. Data are presented as the mean ± SEM. Tadalafil (20 mg/kg) was injected i.p. 30, 60, 90, 120, and 240 min before mice decapitation. Experimental groups consisted of 6–10 animals. Serum and brain concentrations of tadalafil are expressed in ng/ml and ng/g, respectively

Tadalafil (kindly provided by Polpharma S.A., Starogard Gdański, Poland), clonazepam (Clonazepamum, Polfa, Warsaw, Poland), oxcarbazepine (Trileptal, Novartis, Warsaw, Poland), tiagabine (Gabitril, Sanofi Winthrop, Gentilly, France), carbamazepine (kindly provided by Polpharma S.A., Starogard Gdański, Poland), and topiramate (Topamax, Janssen-Cilag International NV, Beerse, Belgium) were suspended in a 1% solution of Tween 80 (POCH, Gliwice, Poland). Valproate (as sodium salt, Sigma-Aldrich) was dissolved in normal saline. The pretreatment time for tadalafil (90 min) was based on pharmacokinetic study results (see Fig. 1). Tiagabine and valproate were administered 15 min, clonazepam and oxcarbazepine 30 min, while carbamazepine and topiramate 60 min prior to the tests. All drug solutions/suspensions were prepared freshly and administered intraperitoneally (i.p.) at a volume of 0.1 ml per 10 g of body weight. Control animals received respective vehicles only.

### Pharmacokinetic Study of Tadalafil

For pharmacokinetic studies, tadalafil was administered at the highest dose used in behavioral studies, i.e., 20 mg/kg i.p. The mice were killed by decapitation 30, 60, 90, 120, and 240 min after tadalafil administration. Blood samples of approximately 1 ml were rapidly harvested, collected into Eppendorf tubes, and allowed to clot at room temperature. Subsequently, they were centrifuged at 3000×*g* for 10 min and serum was collected into polyethylene tubes. Simultaneously, the brains were removed from skulls and washed with 0.9% NaCl. The samples were kept at − 25 °C until analysis.

Tadalafil was measured in mouse serum (100 μl) and brain homogenate (1 ml) by a HPLC method with fluorescence detection. Brains were homogenized in distilled water (1:4, *w*/*v*) with a tissue homogenizer TH220 (Omni International, Inc., Warrenton, VA, USA). The extraction from both serum and brain homogenate was performed using dichloromethane. Prior to the extraction, all samples were spiked with agomelatine solution (500 ng/ml) used as an internal standard (IS) and serum samples were alkalized with 0.1 mol/l Na_2_CO_3_, whereas 1 ml of concentrated NaCl (20 g/100 ml) was added to brain homogenates and the samples were vortexed for 15 s. After addition of the extraction reagent, the samples were shaken for 20 min (IKA VXR Vibrax, Germany) and centrifuged at 1000×*g* for 15 min (Universal 32, Hettich, Germany). The organic layers were transferred into conical tubes and evaporated to dryness at 37 °C under a gentle stream of nitrogen. The residue was dissolved in 100 μl of methanol and 20 μl of this solution was injected into the LaChrom Elite HPLC system (Merck Hitachi, Japan) consisting of an L-2130 pump, an L-2200 autosampler, an L-2485 fluorescence detector, and an L-2350 column oven. EZChrome Elite v. 3.2 (Merck Hitachi) software was used for data acquisition. The separation was performed on a LiChrospher® 100RP-18 column (250 × 4 mm, 5 μm) coupled with a LiChroCART guard column (4.0 × 4.0 mm) (Merck, Germany) with the same packing material. The mobile phase consisted of acetonitrile and water mixed at 45:55 (*v*/*v*) ratio and it was pumped at a flow rate of 0.7 ml/min. The column oven temperature was kept at 35 °C. The detector excitation and emission wavelengths were set at 281 and 330 nm, respectively. Under these conditions, retention times were 8 min for tadalafil and 10.1 min for IS. No interfering peaks from biological matrices were observed. The calibration curves were constructed by plotting the peak area ratios of the analyte to IS versus corresponding concentrations of the analyte, and they were linear in the range of tested concentrations. The limit of quantification was 10 ng/ml for serum and 25 ng/g for brain homogenate. The assay was reproducible with low intra- and inter-day variation (CV was less than 10%).

### Intravenous Pentylenetetrazole Seizure Threshold Test

At the appropriate time after drug suspensions or vehicle administration, mice were placed in the cylindrical plastic restrainer (12-cm long, 3-cm inner diameter). The lateral tail vein was catheterized with a 2-cm long 27-gauge needle attached by polyethylene tubing PE20RW (Plastics One Inc., Roanoke, VA, USA) to a 5-ml plastic syringe containing 1% aqueous solution of PTZ (Sigma-Aldrich). The syringe was mounted on a syringe pump (model Physio 22, Hugo Sachs Elektronik–Harvard Apparatus GmbH, March-Hugstetten, Germany). The accuracy of needle placement in the vein was confirmed by appearance of blood in the tubing. The needle was secured to the tail by an adhesive tape. Following catheterization, mice were released from the restrainer and placed in a Plexiglas arena for behavioral observation. The PTZ solution was infused at a constant rate of 0.2 ml/min. The time intervals from the commencement of PTZ infusion to the onset of each of three endpoints (the first myoclonic twitch, generalized clonus with loss of the righting reflex, and tonic forelimb extension) were recorded. The PTZ infusion was stopped at the beginning of tonic seizures, which were usually lethal for mice. All surviving animals were euthanized immediately. In case of non-appearance of tonic seizure, the infusion of PTZ was terminated at 180 s. For such animals, the dose of PTZ in mg/kg infused till 180 s was taken as the threshold dose. The seizure thresholds were calculated separately for each endpoint using the following formula, threshold dose of PTZ (mg/kg) = (infusion duration (s) × infusion rate (ml/s) × PTZ concentration (mg/ml) × 1000)/body weight (kg), and were expressed as the dose of PTZ (in mg/kg) needed to produce the first apparent sign of each endpoint. Data obtained in the i.v. PTZ seizure threshold test were presented as the amount of PTZ (in mg/kg) ± SEM needed to produce the first apparent sign of each endpoint in each experimental group.

### Maximal Electroshock Seizure Test

Constant current stimuli (sine-wave pulses at 50 Hz for 200 ms) were applied via saline-soaked transcorneal electrodes with the usage of rodent shocker (type 221; Hugo Sachs Elektronik, Freiburg, Germany). During stimulation, mice were restrained manually and immediately following stimulation were placed in a transparent box for behavioral observation for the presence or absence of seizure activity. Tonic hindlimb extension, defined as the rigid extension of the hindlimb that exceeds a 90° angle with the body, was taken as an endpoint. Two experimental approaches to induce electroconvulsions were used in the present study: (1) the maximal electroshock seizure threshold (MEST) test that employed stimulation at varied current intensities (7.6–17.4 mA) and (2) the maximal electroshock seizure (MES) test that employed stimulation at a fixed current intensity (25 mA).

In the MEST test, the current intensity was established according to an “up-and-down” method described by Kimball et al. (1957). Current intensity was lowered or raised by 0.06-log intervals depending on whether the previously stimulated animal did or did not exert tonic hindlimb extension, respectively. The data obtained in groups of 20 animals were used to determine the threshold current causing endpoint in 50% of mice (CS_50_ with confidence limits for 95% probability).

The MES test was performed in order to evaluate the anticonvulsant potency of carbamazepine and topiramate administered alone or in combination with tadalafil. The animals (3–4 groups, 8 animals/group) were injected with increasing doses of antiepileptic drugs or their combinations with tiagabine and then challenged with supramaximal MES stimulus (25 mA). The percentage of mice failing to achieve hindlimb extension was noted, and a log-probit method (Litchfield and Wilcoxon 1949) was used to determine the median effective doses (ED_50_) of antiepileptic drugs, i.e., doses (in mg/kg) that protect 50% of animals.

### Six-Hertz Psychomotor Seizure Test

Square-wave alternating current stimuli (0.2-ms duration pulses at 6 Hz for 3 s) were applied via saline-soaked corneal electrodes using a Grass model CCU1 constant current unit coupled to a Grass S48 stimulator (Grass Technologies, Warwick, RI, USA). Before stimulation, the corneal electrodes were wetted with saline to provide good electrical contact. Mice were manually restrained during stimulation. Immediately following the stimulation, mice were placed in a transparent box for behavioral observation. Psychomotor seizures were characterized by stun (fixed) posture, rearing, forelimb clonus, twitching of the vibrissae, and elevated tail. Lack of the features listed above or the resumption of normal exploratory behavior within 10 s after stimulation were considered as the absence of seizures. Two experimental approaches to induce psychomotor seizures were used in the present study: (1) the 6-Hz seizure threshold test that employed stimulation at varied current intensities (10.0–20.0 mA) and (2) the 6-Hz seizure test that employed supramaximal stimulation at a fixed current intensity at 32 mA (Giordano et al. 2015, 2016).

In the 6-Hz seizure threshold test, the current intensity values were chosen according to an “up-and-down” method (Kimball et al. 1957). Each animal was stimulated only once at any given current intensity that was lowered or raised by 0.06-log intervals depending on whether the previously stimulated animal did or did not respond with convulsions. The data obtained in groups of 20 animals were used to determine the threshold current causing 6-Hz-induced seizures in 50% of mice (CS_50_ with confidence limits for 95% probability).

The 6-Hz seizure test at fixed current intensity of 32 mA was used to determine the protective potency of valproate and tiagabine. Mice (3–4 groups, 8 animals/group) were injected with increasing doses of antiepileptic drugs or their combinations with tadalafil and then stimulated with supramaximal current intensity of 32 mA. As in the MES test, a log-probit method (Litchfield and Wilcoxon 1949) was used to determine the ED_50_ values of antiepileptic drugs in this test.

### Grip-Strength Test

The acute effects of tadalafil, antiepileptic drugs, and their combinations with tadalafil on skeletal muscular strength in mice were quantified in the grip-strength test. The grip-strength apparatus (BioSeb, Chaville, France) consisted of a steel wire grid (8 × 8 cm) connected to an isometric force transducer. The animal was lifted by its tail so that it could grasp the grid with its forepaws. The mouse were then gently pulled backward until it released the grid and the maximal force in newtons (N) exerted by the mouse before losing grip was measured. The procedure was repeated three times and the mean force exerted by each mouse before losing grip was recorded. The mean force was then normalized to body weight and expressed in mN/g ± SE.

### Chimney Test

The chimney test was used to detect the motor deficits in mice induced either by tadalafil, antiepileptic drugs, or combinations of tadalafil with the studied antiepileptic drugs. In this test, the inability of animals to climb backward up through a Plexiglas tube (3 cm, inner diameter, × 30 cm, length) within 60 s is an indicator of motor impairment.

### Step-Through Passive Avoidance Test

The step-through passive avoidance test was used for testing the effect of antiepileptic drugs and their combination with tadalafil on long-term memory impairments in mice. On the first day before training, each animal was administered with antiepileptic drug or its combination with tadalafil and placed in the illuminated box (10 × 13 × 15 cm) connected via a guillotine door to the dark box (25 × 20 × 15 cm) with an electric grid floor. Entrance of the animal to the dark box was punished by an adequate electric shock (0.6 mA foot shock for 2 s). Twenty-four hours later, animals were placed again into illuminated box and observed for up to 180 s. Mice that avoided dark part were recognized to remember the task. The retention time (the time that the mice took to enter to the dark box) was noted and the results were presented as median latencies (retention times; in seconds) with 25th and 75th percentiles.

### Determination of Clonazepam and Oxcarbazepine Concentrations

Thirty minutes following administration of clonazepam or oxcarbazepine with or without tadalafil, mice were decapitated to collect biological material for drug concentration measurement in pharmacokinetic studies. Tadalafil was injected 90 min before decapitation. Blood was collected into Eppendorf tubes and allowed to clot at room temperature. Subsequently, it was centrifuged 3000×*g* for 10 min and serum was collected into polyethylene tubes. Immediately after the decapitation, brains were dissected from the skull and washed with 0.9% NaCl. The samples were kept at − 25 °C until analysis.

Plasma and brain concentrations of clonazepam were measured by a liquid chromatography tandem mass spectrometry (LC-MS/MS) method. A simple liquid-liquid extraction with a mixture of ethyl acetate:hexane (30:70, *v*/*v*) was used to isolate clonazepam from serum (200 μl) or brain homogenate (1 ml) in water (1:4, *w*/*v*). To each sample containing carbamazepine (50 ng/ml) as an IS, 5 ml of extraction solvent was added and the samples were vigorously shaken for 20 min (IKA VXR Vibrax, Germany). After centrifugation (1000×*g* for 15 min), the organic layers were transferred into conical tubes and evaporated to dryness at 37 °C under a stream of nitrogen. The residue was dissolved in 100 μl of mobile phase and 15 μl of this solution was injected onto the column. The HPLC system (Agilent 1100, Agilent Technologies, Waldbronn, Germany) consisted of a degasser, binary pump, column oven, and an autosampler. Chromatographic separation was carried out on XBridge™ C18 analytical column (3 × 50mm, 5 μm, Waters, Ireland) with the oven temperature set at 30 °C. The mobile phase containing 0.1% formic acid in acetonitrile (A) and 0.1% formic acid in water (B) was set at a flow rate of 0.4 ml/min. Initial mobile phase composition was 95% B with a linear gradient to 20% B in the first 5 min, then isocratic mode for 5 min with the following rapid change back to 95% B in 0.1 min. The remaining time of elution was set at 95% B. The whole HPLC operation lasted 13 min. Mass spectrometric detection was performed on an Applied Biosystems MDS Sciex (Concord, Ontario, Canada) API 2000 triple quadrupole mass spectrometer equipped with an electrospray ionization (ESI) interface. ESI ionization in the positive ion mode was used for the ion production. The tandem mass spectrometer was operated at unit resolution in the selected reaction monitoring (SRM) mode, monitoring the transition of the protonated molecular ions m/z 316 to 270 and m/z 316 to 214 for clonazepam (first pair was used as an quantifier and the second for the identity verification—qualifier) and m/z 237 to 194 for carbamazepine used as an IS. The mass spectrometric conditions were optimized for clonazepam by continuous infusion of the standard solution at the rate of 10 μl/min using a Harvard infusion pump. The ion source temperature was maintained at 450 °C. The ionspray voltage was set at 5500 V. The curtain gas (CUR) was set at 30 and the collision gas (CAD) at 2. The optimal collision energy (CE) was 43 V. The following parameters of ion path were used as the most favorable ones: declustering potential (DP) at 26 V, focusing potential (FP) at 330 V, and entrance potential (EP) at 4 V. Data acquisition and processing were accomplished using the Applied Biosystems Analyst version 1.6 software. The calibration curves were constructed by plotting the ratio of the peak area of the studied drug to IS versus drug concentration and generated by weighted (1/x) linear regression analysis. The validated quantitation ranges for this method were within the expected concentration ranges, namely from 1 to 200 ng/ml for serum and 1–100 ng/g for brain tissue with accuracy from 88.48 to 110.97% and from 88.29 to 106.51% for serum and brain, respectively. The assays were reproducible with low intra- and inter-day variation (coefficient of variation less than 15%). No significant matrix effect was observed and there were no stability-related problems during the routine analysis of samples.

In order to determine oxcarbazepine concentrations, 100 μl of serum sample diluted with 100 μl of water and 200 μl of brain homogenate (1:4, *w*/*v*) in water were spiked with carbamazepine (1 μl/ml) employed as an IS. To brain homogenates, NaCl solution (10 g/100 ml) was added at a ratio of 1:1 and the samples were vortexed for 15 s. Subsequently, 1 ml of ethyl acetate was added to both serum samples and brain homogenates and vigorously shaken for 20 min (IKA VXR Vibrax, Germany). After centrifugation (TDX Centrifuge, Abbott Laboratories, USA), the organic layers of brain samples were transferred into conical tubes and evaporated to dryness at 37 °C under a stream of nitrogen. The organic layers separated from serum samples were washed with 1 ml of 1 M HCl before evaporation. The residues were dissolved in 100 μl of acetonitrile and 5 μl of these solutions was injected into the HPLC system. The HPLC system (Merck Hitachi, Darmstadt, Germany) consisted of an L-7100 isocratic pump, an L-7200 autosampler, and a UV variable-wavelength K-2600 detector (Knauer, Berlin, Germany). D-7000 HSM software was used for data acquisition and processing. Analysis was performed on a LiChrospher® 100RP-18 column (250 × 4 mm, 5 μm) coupled with a LiChroCART guard column (4.0 × 4.0 mm) (Merck, Germany) with the same packing material. The mobile phase consisting of acetonitrile and water (36:64, *v*/*v*) was pumped at a flow rate of 1 ml/min. Chromatographic analyses were carried out at room temperature and the analytical wavelength was 210 nm. In these conditions, the retention times were 5.5 min for oxcarbazepine and 9.1 min for IS. The calibration curves were constructed by plotting the ratio of the peak heights of the studied drug to IS versus drug concentration, and they were linear in the tested concentration range. The relative error for accuracy and the coefficient of variation for precision were less than 10%. No interfering peaks were observed in the blank serum or brain homogenate and tadalafil did not interfere with the assay.

### Statistical Analysis

Data from seizure tests as well as from the grip-strength test were analyzed using one-way analysis of variance (one-way ANOVA) followed by the Tukey’s post hoc test. The Fisher’s exact probability test was employed to compare the data from the chimney test. The results obtained in the passive avoidance task were compared with the Kruskal-Wallis test. Serum and brain concentrations of clonazepam and oxcarbazepine were analyzed using the unpaired Student’s *t* test.

*p* < 0.05 was considered statistically significant with 95% confidence (GraphPad Prism version 5.03 for Windows, GraphPad Software, San Diego, CA, USA).

## Results

### Brain and Serum Concentrations of Tadalafil

Brain and serum concentrations of tadalafil after its acute administration (at a dose of 20 mg/kg i.p.) are shown in Fig. 1. The highest serum concentration of tadalafil was noted 90 min after its injection. Likewise, tadalafil reached a peak brain concentration at 90 min after administration.

### Effect of Tadalafil on the Seizure Threshold in the i.v. PTZ Test in Mice

**Fig. 2:**
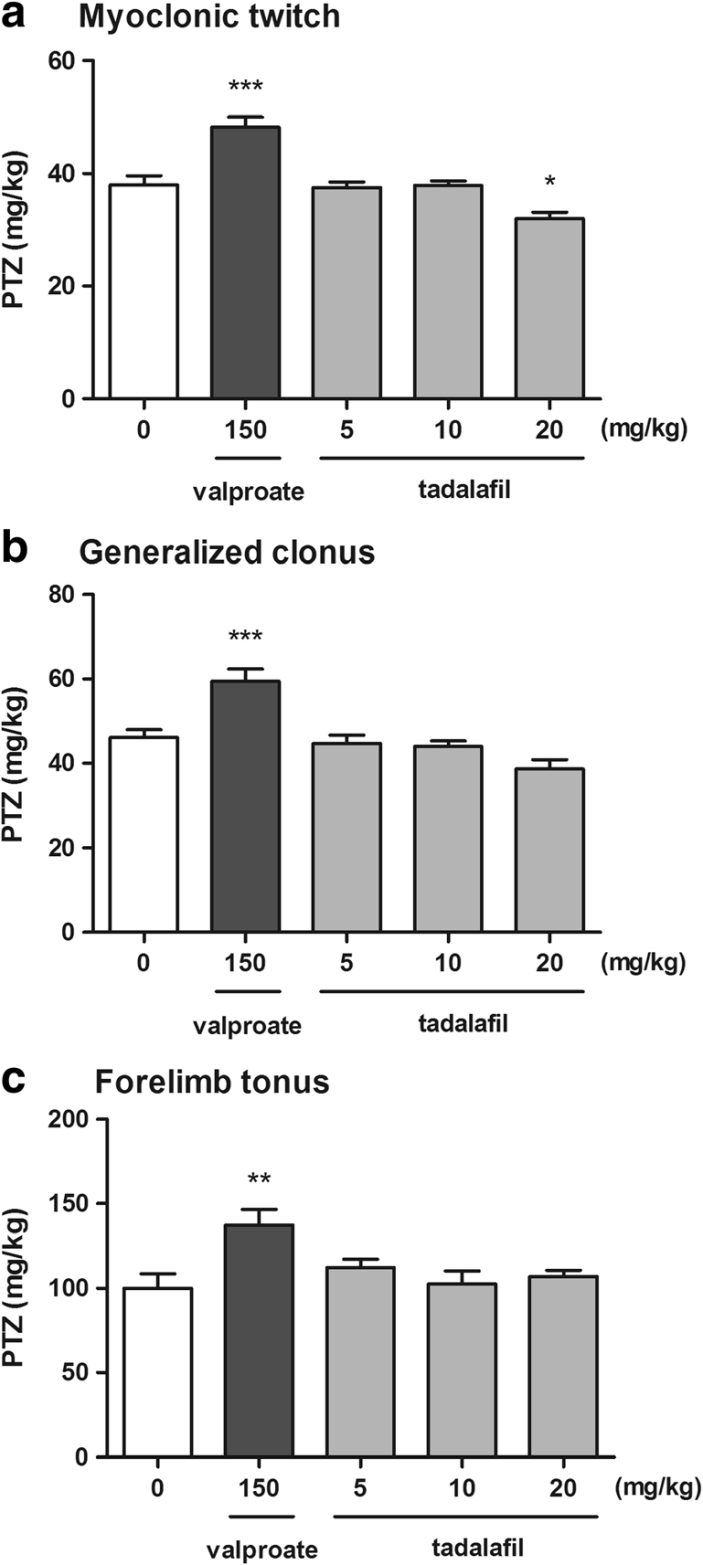
Effect of tadalafil on the threshold for the first myoclonic twitch (**a**), generalized clonus (**b**), and forelimb tonus (**c**) in the i.v. PTZ seizure threshold test in mice. Tadalafil and valproate (positive control) were administered i.p. 90 and 15 min before the test, respectively. The doses are shown on the abscissa. Control animals received 1% Tween 80. Each experimental group consisted of 10–13 animals. Each bar represents the mean (mg/kg PTZ) + SEM. *p < 0.05, ***p < 0.001 versus the control group (one-way ANOVA followed by the Tukey’s post hoc test)

The effect of tadalafil on the seizure threshold in the i.v. PTZ test is shown in Fig. 2a–c (one-way ANOVA: *F*(4,50) = 17.98, *p* < 0.0001 for myoclonic twitch; *F*(4,49) = 12.98, *p* < 0.0001 for generalized tonus; *F*(4,45) = 4.34, *p* = 0.005 for forelimb tonus). Tadalafil administered at doses of 5 and 10 mg/kg did not significantly affect the susceptibility of mice to the PTZ-induced first myoclonic twitch. However, at the highest dose tested, 20 mg/kg, it significantly decreased the threshold for the first myoclonic twitch (Tukey’s post hoc test: *p* < 0.05 vs. control group). No statistically significant changes in the thresholds for the onset of generalized clonic seizures with loss of righting reflex and forelimb tonic extension after tadalafil (5–20 mg/kg) administration were observed. In contrast, valproate (a positive control) at a dose of 150 mg/kg significantly increased the threshold for the onset of all the studied endpoints (Tukey's post hoc test: *p* < 0.001 for myoclonic twitch and generalized clonus; *p* < 0.01 for forelimb tonus).

### Effect of Tadalafil on the Seizure Threshold in the MEST Test

**Fig. 3:**
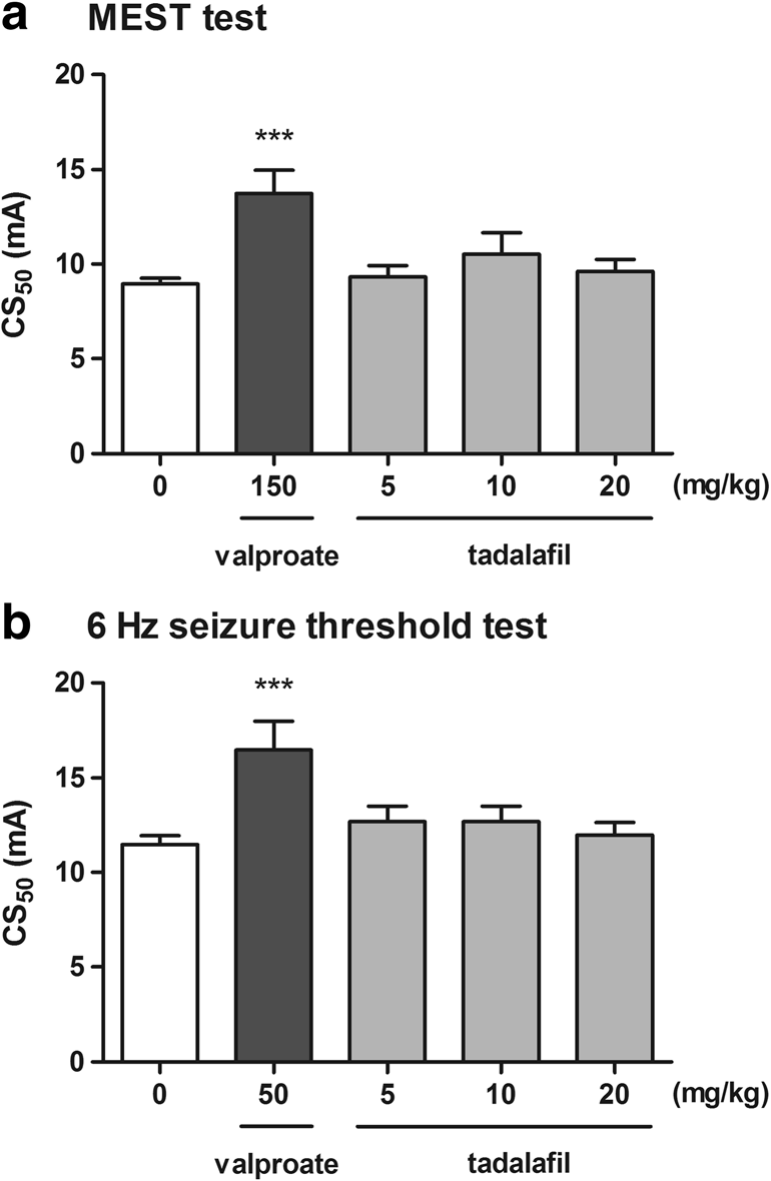
Effect of tadalafil on the seizure threshold in the MEST (**a**) and the 6-Hz-induced seizure test (**b**). Tadalafil and valproate (positive control) were administered i.p. 90 and 15 min before the test, respectively. The doses are shown on the abscissa. Control animals received 1% Tween 80. Each experimental group consisted of 20 animals. Data are presented as CS_50_ (in mA) values with upper 95% confidence limits. Each CS_50_ value represents current intensity predicted to produce convulsions in 50% of mice. ***p < 0.001 versus the control group (one-way ANOVA followed by the Tukey’s post hoc test)

The influence of tadalafil on the threshold for the tonic hindlimb extension in the MEST test is shown in Fig. 3a (one-way ANOVA: *F*(4,42) = 25.05, *p* < 0.0001). Tadalafil administered at doses of 5, 10, and 20 mg/kg had no significant effect on the CS_50_ value in the MEST test. Positive control (valproate at 150 mg/kg) produced a significant increase in the threshold for the hindlimb extension (Tukey’s post hoc test: *p* < 0.001 vs. the control group).

### Effect of Tadalafil on the Seizure Threshold in the 6-Hz Seizure Threshold Test

Figure 3b presents the effect of tadalafil on the threshold for psychomotor seizure in the 6-Hz seizure test (one-way ANOVA: *F*(4,42) = 30.34, *p* < 0.0001). The threshold for the 6-Hz-induced psychomotor seizure was not affected by tadalafil administered at doses of 5, 10, and 20 mg/kg. In comparison, positive control (valproate at 50 mg/kg) produced a significant increase in the psychomotor seizure threshold (Tukey’s post hoc test: *p* < 0.001).

### Effect of Tadalafil on the Activity of Clonazepam and Oxcarbazepine in the i.v. PTZ Seizure Threshold Test

**Fig. 4:**
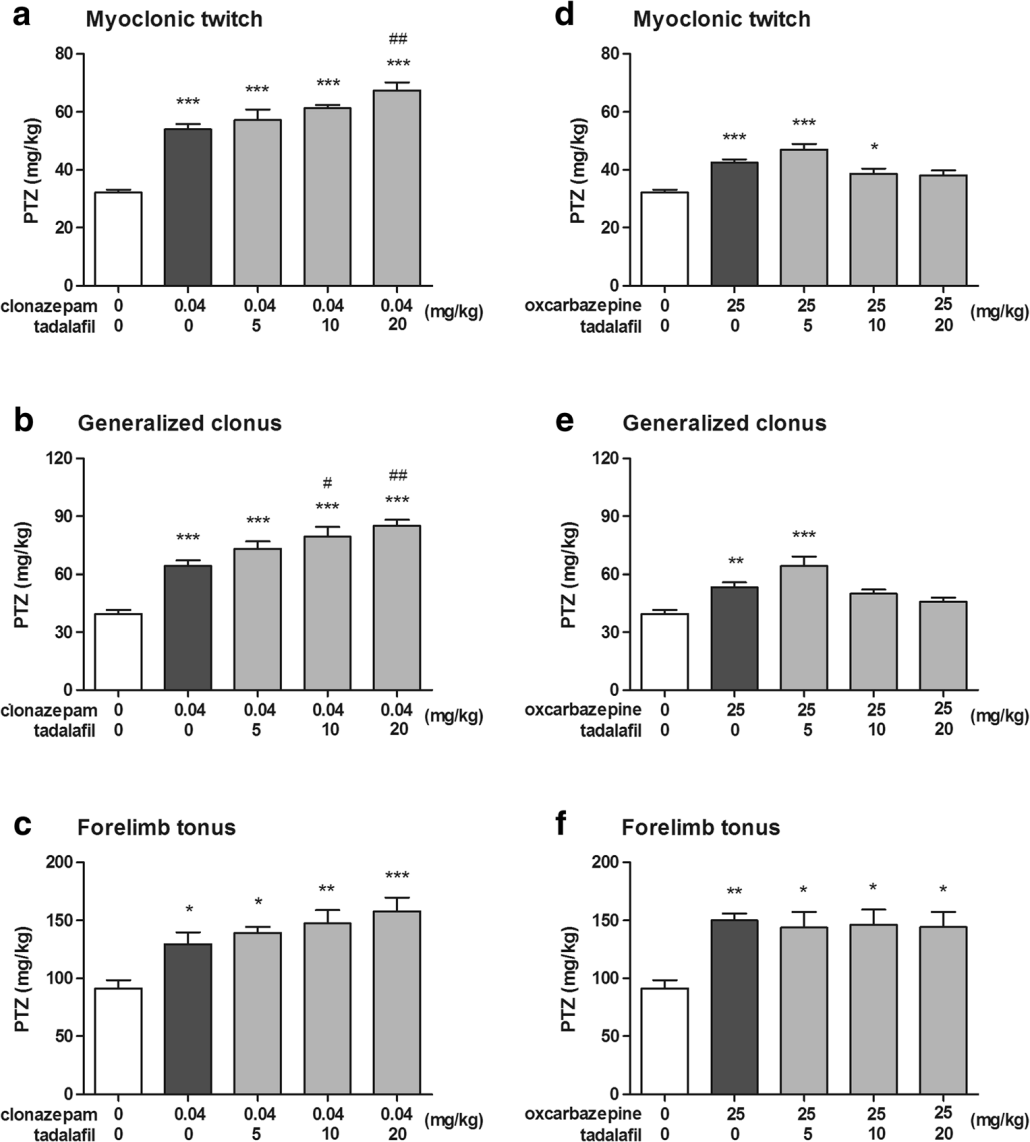
Effect of clonazepam and oxcarbazepine administered alone and in combination with tadalafil on the threshold for the first myoclonic twitch (**a**, **d**), generalized clonus (**b**, **e**), and forelimb tonus (**c**, **f**) in the i.v. PTZ seizure threshold test in mice. Tadalafil was administered 90 min, while clonazepam and oxcarbazepine 30 min before the test. All the drugs were injected i.p. The doses are shown on the abscissa. Control animals received vehicles only. Each experimental group consisted of 8–13 animals. Each bar represents the mean (mg/kg PTZ) + SEM. *p < 0.05, **p < 0.01, ***p < 0.001 versus the control group; ^#^*p* < 0.05, ^##^*p* < 0.01 versus the clonazepam-treated group (one-way ANOVA followed by the Tukey’s post hoc test)

Effect of clonazepam administered alone as well as in combination with tadalafil on the threshold for the first myoclonic twitch in the i.v. PTZ test (one-way ANOVA: *F*(4,52) = 33.17, *p* < 0.0001) is shown in Fig. 4a. Clonazepam injected alone at a dose of 0.04 mg/kg significantly increased the PTZ threshold for the first myoclonic twitch (Tukey’s post hoc test: *p* < 0.001 vs. the control group). Co-administration of tadalafil at doses of 5 and 10 mg/kg did not cause any additional effects on the threshold for myoclonic seizure as compared to clonazepam alone. When injected a higher dose (20 mg/kg), tadalafil augmented the anticonvulsant effect of clonazepam (Tukey’s post hoc test: *p* < 0.01 vs. the clonazepam-treated group).

Effect of clonazepam administered alone as well as in combination with tadalafil on the threshold for the onset of the generalized clonus in the i.v. PTZ test (one-way ANOVA: *F*(4,52) = 22.59, *p* < 0.0001) is shown in Fig. 4b. Clonazepam injected alone at a dose of 0.04 mg/kg significantly increased the PTZ threshold for generalized clonic seizure (Tukey’s post hoc test: *p* < 0.001 vs. the control group), while co-administration of tadalafil at doses of 10 and 20 mg/kg significantly potentiated the anticonvulsant activity of clonazepam against clonic seizure (Tukey’s post hoc test: *p* < 0.05 and *p* < 0.01 vs. the clonazepam-treated group, respectively). Joint administration of clonazepam and tadalafil at the lowest dose tested, i.e., 5 mg/kg, did not cause any additional effect on the PTZ threshold for generalized clonus as compared to the clonazepam-treated group.

Effect of clonazepam administered alone as well as in combination with tadalafil on the threshold for the onset of forelimb tonus in the i.v. PTZ test (one-way ANOVA: *F*(4,44) = 6.74, *p* < 0.001) is shown in Fig. 4c. Clonazepam injected alone at a dose of 0.04 mg/kg significantly increased the PTZ threshold for the onset of forelimb tonic extension (Tukey’s post hoc test: *p* < 0.05 vs. the control group). Tadalafil injected at doses of 10 and 20 mg/kg caused further increase in the threshold for the PTZ-induced tonic seizure (Tukey’s post hoc test: *p* < 0.01 and *p* < 0.001 vs. the control group, respectively). The observed changes were not, however, statistically significant in comparison to clonazepam alone.

Effect of oxcarbazepine administered alone as well as in combination with tadalafil on the threshold for the onset of the first myoclonic twitch in the i.v. PTZ test (one-way ANOVA: *F*(4,51) = 11.62, *p* < 0.0001) is shown in Fig. 4d. Oxcarbazepine injected alone at a dose of 25 mg/kg significantly raised the threshold for myoclonic twitch (Tukey’s post hoc test: *p* < 0.001 vs. the control group). Statistically significant raise in the threshold for myoclonic seizures was also observed in groups of animals treated with combination of oxcarbazepine and tadalafil at 5 and 10 mg/kg. When given at 10 mg/kg, tadalafil abolished the anticonvulsant effect of oxcarbazepine (*p* > 0.05 as compared to the control group).

Effect of oxcarbazepine administered alone as well as in combination with tadalafil on the threshold for the onset of the generalized clonus in the i.v. PTZ test (one-way ANOVA: *F*(4,51) = 10.20, *p* < 0.0001) is shown in Fig. 4e. Oxcarbazepine administered alone and in combination with tadalafil at 5 mg/kg significantly raised the threshold for generalized clonic seizure (*p* < 0.01 and *p* < 0.001, respectively). No such effect was observed when oxcarbazepine was co-administered with tadalafil at doses of 10 and 20 mg/kg (*p* > 0.05 vs. the control group).

Effect of oxcarbazepine administered alone as well as in combination with tadalafil on the threshold for the onset of forelimb tonus in the i.v. PTZ test (one-way ANOVA: *F*(4,47) = 4.50, *p* = 0.004) is shown in Fig. 4f. Oxcarbazepine injected alone significantly increased the threshold for the PTZ-induced forelimb tonus (*p* < 0.01 vs. control group). Co-administration of tadalafil at all doses tested did not affect the anticonvulsant potency of oxcarbazepine against tonic forelimb extension (*p* < 0.05 vs. control group).

### Effect of Tadalafil on the Activity of Carbamazepine and Topiramate in the MES Test

**Fig. 5:**
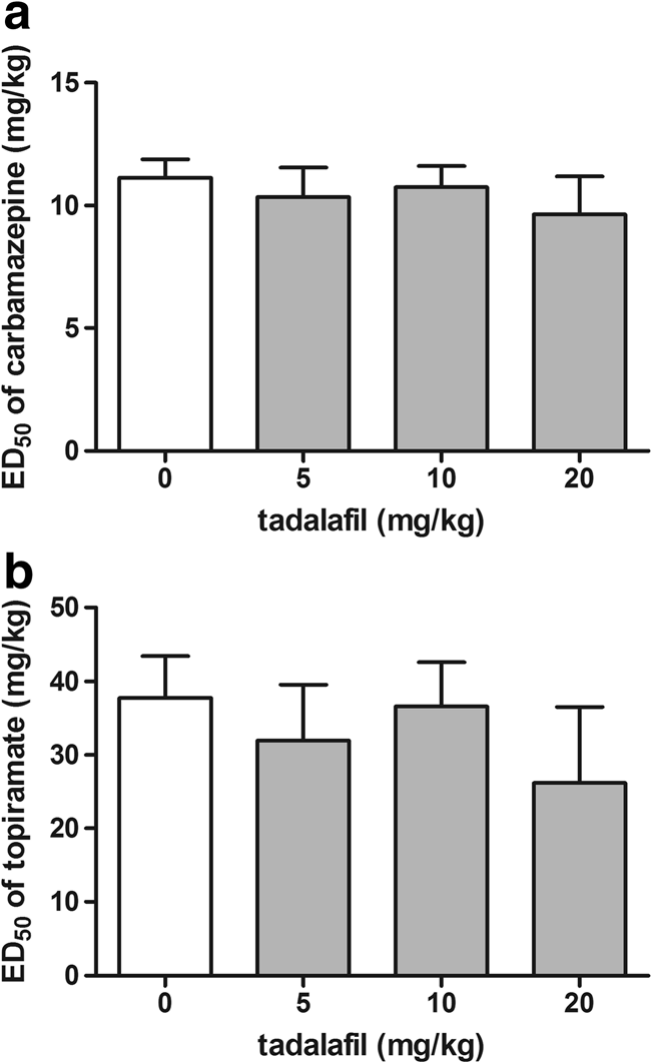
Effect of tadalafil on the anticonvulsant potency of carbamazepine (**a**) and topiramate (**b**) in the MES test in mice. Tadalafil was administered 90 min, carbamazepine 30 min, and topiramate 60 min prior to the test. All the drugs were injected i.p. Control animals received an antiepileptic drug and 1% Tween 80 instead of tadalafil. Each experimental group consisted of 24–32 animals. Each ED_50_ (+ SEM) value represented a dose of an antiepileptic drug predicted to protect 50% of mice tested against maximal electroshock-induced convulsions

Effect of tadalafil on the anticonvulsant potency of carbamazepine and topiramate in the MES test is shown in Fig. 5a, b (one-way ANOVA: *F*(3,100) = 0.25, *p* = 0.862 for panel a and *F*(3,76) = 0.303, *p* = 0.823 for panel b). Carbamazepine and topiramate administered alone produced anticonvulsant effect against maximal electroshock-induced seizure. Tadalafil injected at doses of 5, 10, and 20 mg/kg had no significant effect on the anticonvulsant action of carbamazepine and topiramate in the MES test in mice.

### Effect of Tadalafil on the Activity of Valproate and Tiagabine in the 6-Hz Seizure Test

**Fig. 6:**
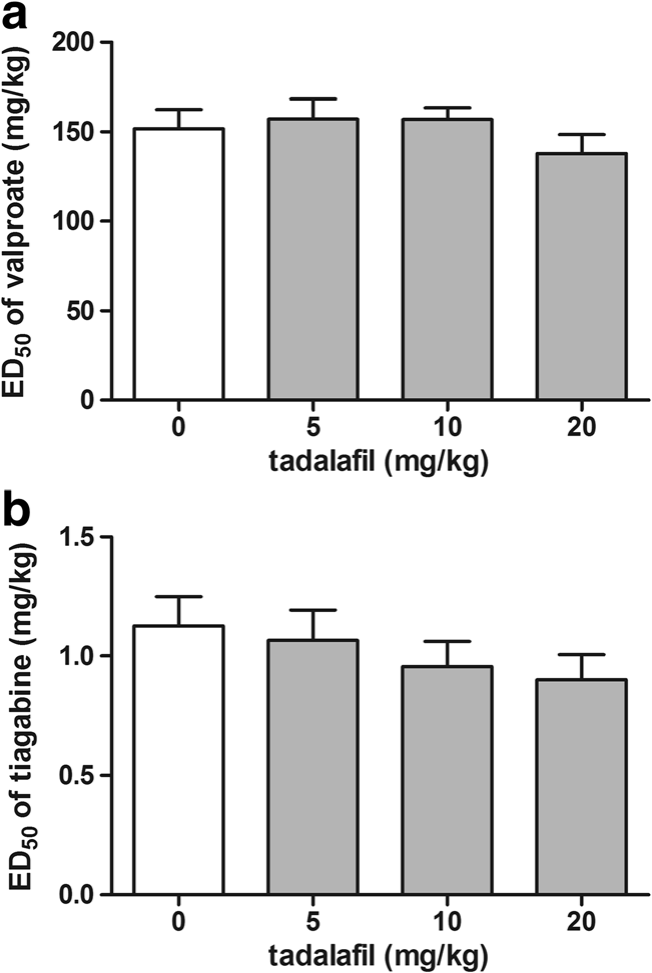
Effect of tadalafil on the anticonvulsant potency of valproate (**a**) and tiagabine (**b**) in the 6-Hz seizure test in mice. Tadalafil was administered 90 min, while valproate and tiagabine 15 min prior to the test. All the drugs were injected i.p. Control animals received an antiepileptic drug and 1% Tween 80 instead of tadalafil. Each experimental group consisted of 24 animals. Each ED_50_ (+ SEM) value represented a dose of an antiepileptic drug predicted to protect 50% of mice tested against 6-Hz-induced psychomotor seizure

The influence of tiagabine on the anticonvulsant potency of valproate and tiagabine in the 6-Hz seizure test is shown in Fig. 6a, b (one-way ANOVA: *F*(3,92) = 0.83, *p* = 0.481 for panel a and *F*(3,60) = 0.84, *p* = 0.477 for panel b). Both valproate and tiagabine administered alone exhibited anticonvulsant activity against the 6-Hz-induced seizure. Tadalafil injected at doses of 5, 10, and 20 mg/kg did not affect significantly the ED_50_ values of both valproate and tiagabine in the 6-Hz seizure test.

### Effects of Tadalafil on Motor Coordination and Muscular Strength in Mice

The acute effects of tadalafil (5–20 mg/kg) on the neuromuscular strength and motor coordination in mice are shown in Table 1. There were no significant impairments of motor coordination in the chimney test (Fisher’s exact test: *p* > 0.05). Likewise, tadalafil at any dose tested did not significantly affect the neuromuscular strength in mice, as determined in the grip-strength test (one-way ANOVA: *F*(4,44) = 0.36, *p* = 0.839).

**Table 1:** Effect of tadalafil on motor coordination and neuromuscular strength in mice

Treatment (mg/kg)	Impairment of motor performance (%)	Neuromuscular strength (mN/g)
Control	0	26.53 ± 0.64
Valproate (150)	0	26.19 ± 1.09
Tadalafil (5)	0	26.05 ± 1.09
Tadalafil (10)	0	26.95 ± 0.45
Tadalafil (20)	10	27.45 ± 1.25

Data are presented as a percentage of animals showing motor coordination impairment in the chimney test and as means ± SEM grip strengths in millinewtons per gram of mouse body weight (mN/g) from the grip-strength test, assessing skeletal muscular strength in mice. Each experimental group consisted of 10 animals. The results from the grip-strength test were analyzed with one-way ANOVA. Statistical analysis of data from the chimney test was performed with the Fisher’s exact probability test

### Effects of Tadalafil in Combination with Antiepileptic Drugs on Muscular Strength, Motor Coordination, and Long-Term Memory in Mice

Neither antiepileptic drugs alone nor their combinations with tadalafil at a dose of 20 mg/kg affected significantly motor coordination, as determined in the chimney test (Fisher’s test: *p* > 0.05 for all experimental groups; Table 2, column I). There were also no significant changes in neuromuscular strength, as assessed in the grip-strength test (one-way ANOVA: *F*(4,44) = 2.455, *p* = 0.060 for group A; *F*(4,45) = 1.113, *p* = 0.362 for group B; *F*(4,45) = 1.628, *p* = 0.184 for group C, Table 2, column II). Likewise, no alterations in long-term memory in mice treated with antiepileptic drugs or their combinations with tadalafil were observed, as assessed in the passive avoidance task (Kruskal-Wallis test: KW = 4.317, *p* = 0.365 for group A; KW = 3.594, *p* = 0.464 for group B; KW = 7.772, *p* = 0.100 for group C; Table 3, column III).

**Table 2:** Effects of antiepileptic drugs and their combinations with tadalafil on motor performance in the chimney test, neuromuscular strength in the grip-strength test, and long-term memory in the passive avoidance task in mice

Treatment (mg/kg)		I. Impairment of motor performance (%)	II. Neuromuscular strength (mN/g)	III. Retention time (s)
A.	Control	0	29.77 ± 0.64	180 (141.8; 180)
	Clonazepam (0.04)	0	31.31 ± 1.49	167 (68.3; 180)
	Clonazepam (0.04) + tadalafil (20)	10	29.06 ± 1.20	165.5 (72; 180)
	Oxcarbazepine (25)	0	30.23 ± 1.16	170 (28.5; 180)
	Oxcarbazepine (25) + tadalafil (20)	0	26.32 ± 1.37	180 (90.5; 180)
B.	Control	0	28.56 ± 0.97	179 (84.75; 180)
	Carbamazepine (9.65)	0	28.38 ± 1.33	180 (105; 180)
	Carbamazepine (9.65) + tadalafil (20)	0	30.71 ± 1.31	180 (180; 180)
	Topiramate (26.21)	0	27.76 ± 1.61	180 (110.3; 180)
	Topiramate (26.21) + tadalafil (20)	10	27.05 ± 1.22	148 (98.0; 180)
C.	Control	0	29.11 ± 1.90	180 (37.75; 180)
	Valproate (137.96)	0	29.99 ± 1.61	175 (35.75; 180)
	Valproate (137.96) + tadalafil (20)	0	26.01 ± 1.49	180 (180; 180)
	Tiagabine (0.90)	0	25.50 ± 0.92	180 (170; 180)
	Tiagabine (0.90) + tadalafil (20)	0	30.00 ± 2.37	106 (28.5; 180)

Data are presented as a percentage of animals showing motor coordination impairment in the chimney test, as mean ± SEM grip strengths in millinewtons per gram of mouse body weight (mN/g) from the grip-strength test, assessing skeletal muscular strength in mice, and as median retention time (in s; with 25th and 75th percentiles in parentheses) from the passive avoidance task, assessing long-term memory in mice. Each experimental group consisted of 9–10 animals. The results from the grip-strength test were analyzed with one-way ANOVA. Statistical analysis of data from the chimney test was performed with the Fisher’s exact probability test. Non-parametric Kruskal-Wallis ANOVA test was used to analyze the results from the passive avoidance task

**Table 3:** Effect of tadalafil on serum and brain concentrations of clonazepam and oxcarbazepine

Treatment (mg/kg)	Serum	Brain
Clonazepam (0.04)	3.96 ± 0.68 ng/ml	16.36 ± 3.13 ng/g
Clonazepam (0.04) + tadalafil (20)	5.23 ± 0.56 ng/ml	18.86 ± 1.09 ng/g
Oxcarbazepine (25)	10.27 ± 0.70 μg/ml	10.13 ± 0.47 μg/g
Oxcarbazepine (25) + tadalafil (20)	10.27 ± 0.48 μg/ml	10.79 ± 0.69 μg/g

Tadalafil was administered 90 min while clonazepam and oxcarbazepine were administered 30 min before the test. Data are presented as means ± SEM. Each experimental group consisted of 8–10 animals. Serum and brain concentrations of antiepileptic drugs were analyzed with the Student’s *t* test

### Effect of Tadalafil on Clonazepam and Oxcarbazepine Concentrations

The influence of tadalafil on serum and brain concentrations of clonazepam and oxcarbazepine is shown in Table 3. Co-administration of tadalafil at a dose of 20 mg/kg and clonazepam at a dose of 0.04 mg/kg did not change clonazepam concentrations in serum and brain tissue (Student’s *t* test: *p* = 0.164 and *p* = 0.460, respectively). Likewise, no changes in serum and brain concentrations of oxcarbazepine in mice co-administered with tadalafil at 20 mg/kg and oxcarbazepine at 25 mg/kg were observed (Student’s *t* test: *p* = 0.996 and *p* = 0.442, respectively).

## Discussion

Although tadalafil was developed initially to act as a vasodilator in peripheral tissues, it also exerts central effects because of its ability to cross the blood-brain barrier and the presence of PDE5 in different brain regions (Peixoto et al. 2015). The involvement of the NO/cGMP/PDE5 pathway in the pathophysiology of epilepsy (Ferraro and Sardo 2004; Riazi et al. 2006) raises the possibility that tadalafil can affect seizure activity, but despite the widespread use of tadalafil, its influence on seizure susceptibility has not been studied. Here, we investigated the effect of acute treatment with tadalafil on seizure thresholds in three different seizure tests in mice that are widely used in antiepileptic drugs research and development (Löscher 2011; Mandhane et al. 2007).

Garcia-Barroso and co-workers (2013) demonstrated for the first time that tadalafil penetrates into the brain after oral administration and it reaches concentrations that may lead to a marked inhibition of PDE5 within different brain structures. In order to characterize the ability of tadalafil to cross the blood-brain barrier after intraperitoneal route of administration in mice, we performed a pilot pharmacokinetic study in which we showed that changes in serum and brain concentrations of tadalafil followed a similar time-course pattern with the maximum concentrations reached at about 90 min post injection. The pretreatment time of 90 min for tadalafil was used in all subsequent experiments to study its acute effects on seizure thresholds. The timed i.v. PTZ infusion test was employed to investigate the effect of tadalafil on chemically induced seizure. This test is considered as extremely sensitive parametric method for assessing seizure thresholds in rodents (White 1998). It is widely accepted that proconvulsant activity of PTZ is at least partially mediated by its ability to block the chloride ion channel in the GABA_A_ receptor complex and the PTZ seizure thresholds are particularly sensitive to compounds that affect GABAergic neurotransmission (Löscher 2009). The obtained results showed that tadalafil did not affect the threshold for the onset of both the generalized clonic seizure with the loss of righting reflex and the tonic forelimb extension in the i.v. PTZ test. Interestingly, tadalafil at the highest dose tested (i.e., 20 mg/kg) significantly decreased the threshold for the first myoclonic twitch. Myoclonus is defined as involuntary, sudden, and brief muscle jerks that are reported in a number of different diseases including myoclonic epilepsy. In addition, myoclonic jerks have been described as an adverse effect of many drugs. The mechanism underlying myoclonus is not fully understood but it is generally considered that alterations of the GABA_A_ receptor activity at different points along the neural axis may contribute to myoclonic jerks (Matsumoto et al. 2000). Of note, GABA_A_ receptor antagonists, such as PTZ or bicuculline, produce myoclonus in animal models (Wheless and Sankar 2003). There is no data on the direct influence of tadalafil on the GABA-mediated transmission. However, several lines of evidence demonstrated the involvement of the NO/cGMP pathway in the GABA-mediated transmission (Prast and Philippu 2001). Since tadalafil acts as a modulator of the NO/cGMP/PDE5 signaling pathway, it is possible that it may affect GABA release and/or GABA_A_ receptor activity. Targeting PDE5 with tadalafil elevates intracellular cGMP level. Accumulation of cGMP may lead to the activation of protein kinase G and subsequent phosphorylation of the GABA_A_ receptor subunits, which results in reduced GABA-mediated neuronal inhibition (Wang and Robinson 1997). It should furthermore be noted that PTZ increases cGMP level in several brain structures (such as cerebral cortex, hippocampus, striatum, and cerebellum), which supports the hypothesis that cGMP plays a role in the initiation and propagation of PTZ-induced seizures (Ferrendelli et al. 1980). Thus, it seems that decreased seizure threshold for myoclonic twitch following tadalafil administration could have resulted from increased cGMP concentration and subsequent decrease in GABA-mediated neuronal inhibition. Additionally, PTZ-induced inhibition of the GABAergic transmission leads to the enhancement of glutamatergic neuronal activity (Watanabe et al. 2013). The glutamate-mediated excitatory transmission seems to be controlled by cGMP in biphasic manner because increased cGMP level suppresses glutamate release while very high cGMP concentrations increase the release of glutamate (Prast and Philippu 2001). Hence, tadalafil at a relatively high dose of 20 mg/kg could have also stimulated the release of excitatory neurotransmitter—glutamate. The possible risk of increased susceptibility to myoclonic seizure after tadalafil treatment as well as the mechanism underlying this phenomenon deserves further investigation.

Despite the fact that tadalafil exerted proconvulsant activity with regard to myoclonic seizures, it did not affect the thresholds for electrically induced seizures. It failed to affect the threshold for both the tonic hindlimb extension and psychomotor seizures in the MEST and the 6-Hz-induced seizure test, respectively. Psychomotor seizures are believed to emanate from the limbic system (Barton et al. 2001), especially from the hippocampus and amygdala (Lucchi et al. 2017; Giordano et al. 2015, 2016). Clonus originates from forebrain structures, whereas tonic seizures involve brainstem (White et al. 2009). The lack of effect of tadalafil on clonic and tonic seizures (in both the i.v. PTZ and the MEST tests) as well as on the psychomotor seizures suggests that the changes in cGMP level were not sufficient to modulate the inhibitory and/or excitatory neurotransmission within the aforementioned brain structures. Alas, the changes in cGMP concentration in different brain areas after tadalafil administration have not yet been determined.

The influence of tadalafil on the efficacy of antiepileptic drugs has not been studied so far. For this reason, we also aimed to evaluate the effect of tadalafil on several first- and second-generation antiepileptic drugs. The obtained results showed that tadalafil did not alter the anticonvulsive activity of carbamazepine and topiramate (against the maximal electroshock-induced seizures) as well as valproate and tiagabine (against the 6-Hz-induced psychomotor seizures). In the i.v. PTZ seizure threshold test, tadalafil potentiated the protective activity of clonazepam against myoclonic and generalized clonic seizures. In contrast, tadalafil decreased the protective activity of oxcarbazepine against PTZ-induced myoclonic and generalized clonic seizures. The interactions between tadalafil and clonazepam or oxcarbazepine may have pharmacodynamic and/or pharmacokinetic basis. Pharmacokinetic interaction occurs when one drug alters the concentration of the other drug by affecting its absorption, distribution, metabolism, or excretion (Corrie and Hardman 2014). To evaluate the potential pharmacokinetic clonazepam-tadalafil and oxcarbazepine-tadalafil interactions, brain and serum concentrations of clonazepam or oxcarbazepine were determined using HPLC methods. No alterations of brain and serum concentrations of the two antiepileptic drugs were noted, which suggests that the changes of their anticonvulsant activity in the i.v. PTZ test were most likely related to pharmacodynamic interactions with tadalafil. Briefly, pharmacodynamic interaction occurs when one drug alters the effect of another drug without changes in its concentration and it involves antagonism or addition of pharmacological properties of the two drugs (Corrie and Hardman 2014). Clonazepam, the first*-*generation antiepileptic drug from the benzodiazepine group, facilitates GABAergic neurotransmission by a direct effect on GABA_A_ receptors. The potentiation of anticonvulsant activity of clonazepam by tadalafil in the i.v. PTZ test is rather an unexpected result because tadalafil was shown to decrease the threshold for myoclonic twitch in this test. Oxcarbazepine belongs to the newer generation of antiepileptic drugs. Its mechanism of action is mostly related to the blockade of voltage-sensitive Na^+^ channels, which reduces high-frequency repetitive firing of neurons. Oxcarbazepine also enhances K^+^ current. Moreover, oxcarbazepine and its active metabolite inhibit high-voltage-activated N-type Ca^2+^ channels and reduce glutamatergic neurotransmission in rat cortex (Łuszczki et al. 2003). There are no direct evidence that tadalafil affects GABAergic and glutamatergic transmission and those ion channels that are involved in initiation, propagation, and amelioration of seizures. We can only speculate that tadalafil (as a NO/cGMP/PDE5 modulator) may modulate inhibitory and excitatory neurotransmission and/or some ion channels functioning. Tadalafil-induced changes in anticonvulsant activity of clonazepam and oxcarbazepine may be a result of their interactions with different cellular targets. Further studies are needed to characterize the basis of these interactions. Other experimental models of seizures or epilepsy should be used as well. Additionally, it would be advisable to test the combination of an ineffective dose of tadalafil with an ineffective dose of clonazepam to confirm the presence of a synergistic interaction between them. Interactions of tadalafil with other benzodiazepines and positive allosteric modulators of the GABA_A_ receptor should be also studied in future.

It is worth mentioning that neither tadalafil alone nor its combinations with the studied antiepileptic drugs produced any acute side effects as determined in the chimney test, the grip-strength test, and the passive avoidance task.

Sildenafil, the first approved PDE5 inhibitor, is the most extensively studied drug from this class. When compared the effects of sildenafil and tadalafil in the three seizure tests employed in the present study, we can see that tadalafil has much weaker influence on the seizure thresholds. It only decreased the seizure threshold for myoclonus twitch in the i.v. PTZ test, whereas sildenafil reduced the threshold for clonic seizure in the i.v. PTZ (Nieoczym et al. 2010b; Riazi et al. 2006), raised the threshold for tonic hindlimb extension in the MEST (Nieoczym et al. 2010a), and raised the threshold for the 6-Hz-induced psychomotor seizure in mice (Nieoczym et al. 2013). Furthermore, the influence of sildenafil and tadalafil on the anticonvulsant activity of antiepileptic drugs is quite different. Specifically, sildenafil raised the ED_50_ value of carbamazepine and topiramate in the MES test (Nieoczym et al. 2010a) as well as valproate and tiagabine in the 6-Hz seizure test (Nieoczym et al. 2013), while tadalafil did not. On the contrary, sildenafil did not affect the anticonvulsant potency of clonazepam in the subcutaneous PTZ test (Nieoczym et al. 2012a) and it increased the activity of oxcarbazepine in the MES test (Nieoczym et al. 2010a). It is also worth mentioning that the interactions of sildenafil with carbamazepine and valproate were shown to be, at least in part, pharmacokinetic in nature (Nieoczym et al. 2010a).

To conclude, our data suggest that tadalafil may increase myoclonic seizure susceptibility but it should not potentially increase the risk of clonic and tonic seizures. Tadalafil may produce some beneficial pharmacodynamics interactions with clonazepam but its co-administration with oxcarbazepine should be rather avoided. However, because our observations have been made in animals, caution should be taken when extrapolating the present results to humans as the response to tadalafil and its influence on the activity of antiepileptic drugs may vary between mice and human subjects. Further studies are required to estimate the risk/benefit ratio of tadalafil usage in epileptic patients.

## Abbreviations

cGMP: Cyclic guanosine 3′,5′-monophosphate
CS_50_: Current strength required to induce seizure response in 50% of mice
ED_50_: Median effective dose
i.p.: Intraperitoneally
IS: Internal standard
i.v.: Intravenously
MES: Maximal electroshock seizure
MEST: Maximal electroshock seizure threshold
N: Newton
PDE5: Phosphodiesterase type 5
PTZ: Pentylenetetrazole
SEM: Standard error of the mean
